# Potential for Distribution Expansion of *Stephanitis chinensis* in China Based on MaxEnt Model

**DOI:** 10.3390/insects17030279

**Published:** 2026-03-04

**Authors:** Hongyan Jiang, Yizhe Wang, Shichun Chen, Shuran Liao, Tingxu Chen, Xiaoqing Wang

**Affiliations:** 1 Tea Research Institute of Chongqing Academy of Agricultural Sciences, Chongqing 402160, China; jianghongyan@cqaas.cn (H.J.); wyizhe202202@163.com (Y.W.); chensc0318@sina.com (S.C.); lsr_717@126.com (S.L.); tx513705@126.com (T.C.); 2 College of Plant Protection, Southwest University, Chongqing 400715, China

**Keywords:** climate change, *Stephanitis chinensis*, MaxEnt model, suitable area, prediction

## Abstract

The tea lace bug, *Stephanitis chinensis*, is an important pest in the southwest tea region in China. It has recently emerged in some parts of the tea areas, severely impacting the profitability of spring tea. To clarify the distribution dynamics of *S. chinensis* under current and future climate change, this study used the MaxEnt model and ArcGIS software to predict the distribution and dominant environmental factors of *S. chinensis*. The results show that the mean precipitation of the warmest quarter (Bio18), the minimum temperature of the coldest month (Bio6), annual precipitation (Bio12), and the variation coefficient of temperature (Bio4) are the dominant environmental factors affecting *S. chinensis* distribution. Under the current climatic conditions, the suitable habitats for *S. chinensis* are mainly distributed in East and South Asia, with only a small distribution in southern Europe, southeastern North America, and coastal areas of southeastern South America; the highly suitable habitats are primarily distributed in China, southern Japan, and southern South Korea. The total suitable area of *S. chinensis* accounts for approximately 28.58% of China’s land area. The high-suitability regions are primarily concentrated in the Guizhou, Chongqing, Sichuan, Hubei, Hunan, Shaanxi, and Jiangsu provinces. Under future climate conditions, the total suitable area of *S. chinensis* will increase to varying degrees, primarily expanding northward, with the extension of high-suitability areas mainly concentrated in Hubei, Anhui, and Henan. The migration distance of the geographical distribution center ranges between 32.27 km and 96.13 km, with a primary shift toward the northeast. This study predicts potential suitable areas for the tea lace bug under different climate change scenarios. Specifically, regions at the highest risk, such as the Hubei, Anhui, and Henan provinces, should enhance monitoring and early warning systems and implement timely prevention and control measures to ensure the safe production of tea.

## Simple Summary

*Stephanitis chinensis* (tea lace bug) is a major pest in China’s southwest tea region, with recent outbreaks severely impacting the profitability of spring tea. To clarify its distribution dynamics under current and future climate change, this study used the MaxEnt model and ArcGIS to predict its distribution and dominant environmental factors. The results show that the key factors are Bio18, Bio6, Bio12, and Bio4. Currently, this species’ suitable habitats are mainly in East and South Asia. In China, suitable habitats account for 28.58% of the total area, with high-suitability regions concentrated in the Guizhou, Chongqing, Sichuan, Hubei, Hunan, Shaanxi, and Jiangsu provinces. Future climate conditions will expand its distribution northward, with the center shifting northeastward. This study provides a basis for monitoring and controlling high-risk regions to ensure tea production safety.

## 1. Introduction

The tea lace bug, *Stephanitis chinensis* (Hemiptera: Tingidae), feeds on tea leaves as both adults and nymphs by piercing and sucking sap, resulting in irreversible whitish stippling on the upper leaf surface. Under heavy infestation, these stipples merge into large patches, and the lower leaf surface becomes covered with abundant black excreta, exuviae, and mold. This damage reduces photosynthetic capacity, induces premature leaf abscission, and weakens overall plant vigor [1,2]. Tea is a major economic crop in China, with approximately 3.43 million hectares under cultivation [3], and it plays an important role in rural economic development and farmers’ livelihoods. The most severe damage caused by *S. chinensis* typically occurs from late March to late May each year [4,5,6], posing a substantial threat to the quality and economic value of spring tea. *S. chinensis* shows strong population persistence [7] and poses risks of both invasive outbreaks and expansions of native populations [5]. Over the past 15 years, the species has exhibited clear patterns of spread and frequent outbreaks, with particularly rapid expansion in recent years in the Shaanxi and Hubei provinces. No distribution records of *S. chinensis* have been reported outside China. Early occurrences were mainly reported in Sichuan, Yunnan, and Guizhou. The species was first detected in the tea-producing area of Zhenba, Hanzhong City, southern Shaanxi, in 2010 [8], and later in Ziyang County, Ankang City, in 2012. By 2016, it had infested 8700 hectares of tea plantations in southern Shaanxi, causing substantial damage and spreading progressively northward [9]. In Hubei, scattered infestations were first reported in 2012 in Longfeng Town, Enshi City. Thereafter, the pest expanded annually and eventually caused major outbreaks. By 2021, its infestation area in the Enshi tea-growing region had reached 14,300 hectares [6]. Given its rapid spread and the extensive cultivation of tea in China, assessing the current and future potential distribution of *S. chinensis* under climate change is essential for understanding its climatic adaptability.

Ecological niche models (ENMs) use species occurrence records combined with environmental variables to simulate a species’ ecological requirements through specific algorithms and then project these requirements across space and time to predict actual and potential distributions [10]. Commonly used ENMs include MaxEnt, CLIMEX, GARP, MARS, and Bioclim.

The MaxEnt model, which is based on the principle of maximum entropy, offers notable advantages over other approaches, particularly when species occurrence data are limited, and it consistently produces robust predictive performance [11]. This model has been widely used to evaluate potential suitable habitats for various species under climate change, including *Corythucha marmorata* [12], *Recaredus rex* [13], and *Empoasca onukii* [14,15]. In recent years, with global climate warming, the occurrence period of tea lace bugs has advanced significantly. To date, no studies have reported on the suitable distribution areas of tea lace bugs. In this study, we used occurrence data of *S. chinensis* together with relevant environmental variables and employed the MaxEnt model integrated with ArcGIS to predict its potential suitable distribution both globally and in China under different climate scenarios. The results are expected to provide a scientific basis for monitoring, early warning, and the development of effective integrated management strategies for *S. chinensis*.

## 2. Materials and Methods

### 2.1. Occurrence Data

**Figure 1:**
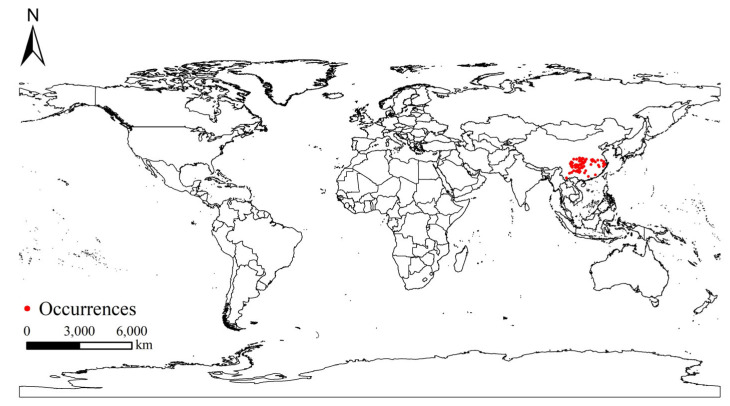
Distribution sites of *Stephanitis chinensis* across the world.

The occurrence data of *S. chinensis* were obtained primarily from the published literature (China National Knowledge Infrastructure and Web of Science Core Collection) and field surveys. A total of 104 occurrence records were compiled (Table S1). To minimize sampling bias and reduce the risk of model overfitting, the spThin package in R 4.2.3 was used to spatially filter the data; when two occurrence points were within 10 km of each other, only one was retained [15,16]. Ultimately, 98 unique occurrence points were used for modeling (Figure 1). The base maps of China and the world were downloaded from the Standard Map Service of the National Administration of Surveying, Mapping and Geoinformation (http://bzdt.ch.mmr.gov.cn/).

### 2.2. Environmental Data

Bioclimatic variables were obtained from the WorldClim 2 database (http://www.worldclim.org/), including current climate data (averaged for 1970–2000) and future projections for 2050 (average for 2041–2060) and 2070 (average for 2061–2080). Nineteen bioclimatic variables were used: annual mean temperature (Bio1), mean diurnal temperature range (Bio2), isothermality (Bio3), temperature seasonality (Bio4), maximum temperature of the warmest month (Bio5), minimum temperature of the coldest month (Bio6), annual temperature range (Bio7), mean temperature of the wettest quarter (Bio8), mean temperature of the driest quarter (Bio9), mean temperature of the warmest quarter (Bio10), mean temperature of the coldest quarter (Bio11), annual precipitation (Bio12), precipitation of the wettest month (Bio13), precipitation of the driest month (Bio14), precipitation seasonality (Bio15), precipitation of the wettest quarter (Bio16), precipitation of the driest quarter (Bio17), precipitation of the warmest quarter (Bio18), and precipitation of the coldest quarter (Bio19). The Shared Socioeconomic Pathways (SSPs) scenario series covers diverse socioeconomic development trends, including population growth, technological advancement, and carbon emission intensity. Compared with the traditional Representative Concentration Pathway (RCP) scenarios, the SSP series is more capable of integrating socioeconomic factors with climatic factors, thereby making their prediction results more practically applicable. Two SSPs were selected for future climate scenarios, namely the sustainability-oriented SSP126 and the high-emission SSP585.

### 2.3. Model Construction

**Figure 2:**
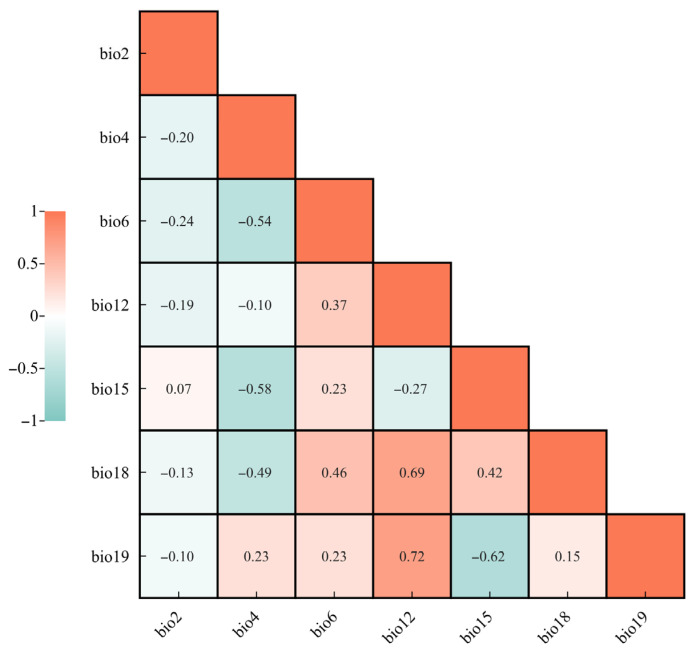
Correlation analysis of major environmental variables.

Environmental variables often exhibit substantial intercorrelation, which can lead to multicollinearity and subsequently reduce model accuracy. To address this issue, we first constructed an initial MaxEnt model using all environmental variables with default settings to obtain the contribution rate of each variable. Variables with a contribution lower than 0.5 were removed. Meanwhile, reverse validation was conducted: after incorporating variables with a contribution rate of <0.5% into the model, there was no improvement in the model’s prediction accuracy (no significant change in the AUC value). The remaining variables associated with the occurrence records of *S. chinensis* were then subjected to Pearson correlation analysis in SPSS 22.0. For any pair of variables with an absolute correlation coefficient greater than 0.8, the variable with the higher contribution rate was retained. Based on the contribution rate and pairwise correlations, seven key bioclimatic variables were ultimately selected from the original nineteen (Figure 2): precipitation of the warmest quarter (Bio18), temperature seasonality (Bio4), minimum temperature of the coldest month (Bio6), precipitation seasonality (Bio15), mean diurnal range (Bio2), precipitation of the coldest quarter (Bio19), and annual precipitation (Bio12).

Parameter optimization was performed using the ENMeval package in R 4.2.3. Six feature combinations—L, H, LQ, LQH, LQHP, and LQHPT—were tested. The regularization multiplier was set from 0.5 to 4.0 in intervals of 0.5, yielding a total of 48 parameter combinations. The Akaike information criterion corrected for small sample size (AICc) was calculated for each combination, and the model with the lowest AICc was considered optimal. Compared with indicators including the AUC difference and omission rate, the AICc better balances model fit and complexity, effectively preventing model overfitting. It is especially suitable for predicting the potential suitable habitats of species with limited sample sizes. The filtered bioclimatic variables and occurrence records were imported into MaxEnt 3.4.1 with 75% of the occurrence points used for training and 25% for testing. Referring to similar studies on the potential suitable habitats of insects, 10,000 background points were established, the cross-validation method was selected, and the maximum number of iterations was set to 5000. Response curves, prediction maps, and the Jackknife test were enabled, and the results were exported in logistic format. The model was run 10 times, and the parameter combination yielding the minimum AICc was selected, while other settings remained at their default values. ArcGIS 10.2 was used to visualize the MaxEnt 3.4.1 outputs and calculate the area of potential suitable habitats with different risk levels.

### 2.4. Model Performance Evaluation

The predictive accuracy of the MaxEnt model was assessed using the omission rate and the area under the receiver operating characteristic curve (AUC). Higher AUC values indicate better model performance. The AUC ranges from 0 to 1; values above 0.7 are generally considered acceptable, values between 0.8 and 0.9 indicate good predictive performance, and values between 0.9 and 1.0 reflect excellent predictive ability [16].

### 2.5. Habitat Suitability Classification and Shifts in Distributional Centroids

The MaxEnt 3.4.1 output was reclassified in ArcGIS 10.2. Based on the predicted probability of occurrence of *S. chinensis*, potential habitat suitability was classified into four categories: unsuitable area (0–0.05), slightly suitable area (0.05–0.3), moderately suitable area (0.3–0.6), and highly suitable area (0.6–1.0). The geographic centroids of each suitability class were calculated using the “Zonal Geometry as Table” tool in ArcGIS.

## 3. Results

### 3.1. Model Optimization and Accuracy Assessment

**Figure 3:**
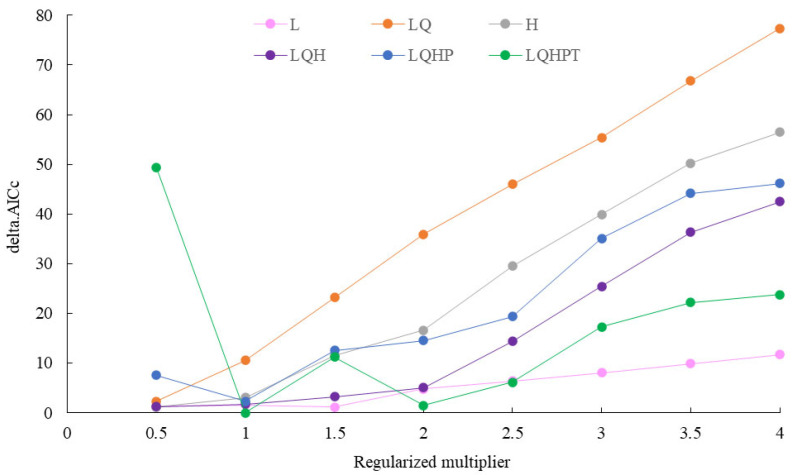
Parameter evaluation curve of the MaxEnt model.

**Figure 4:**
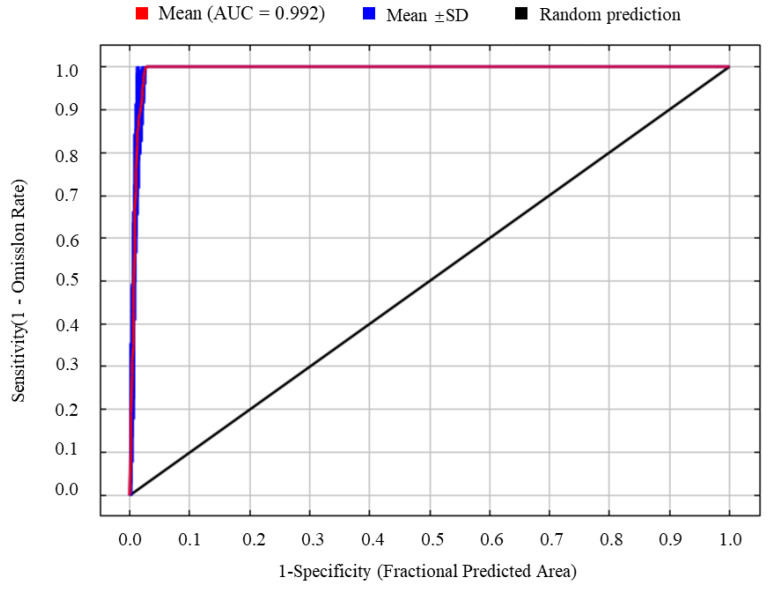
The receiver operating characteristic (ROC) curve generated by the MaxEnt model.

When the regularization multiplier was set to 1 and the feature combination was LQHPT, the model produced the lowest AICc value, indicating that this parameterization yielded optimal model performance (Figure 3). Using the geographic coordinates of 98 occurrence records of *S. chinensis* and seven selected environmental variables, the MaxEnt model was run ten times. The resulting AUC value was 0.992 (Figure 4), representing “excellent” predictive accuracy. This demonstrates that the model provides highly reliable and accurate predictions of the potential distribution of *S. chinensis*.

### 3.2. Dominant Environmental Variables Influencing the Distribution of S. chinensis

**Figure 5:**
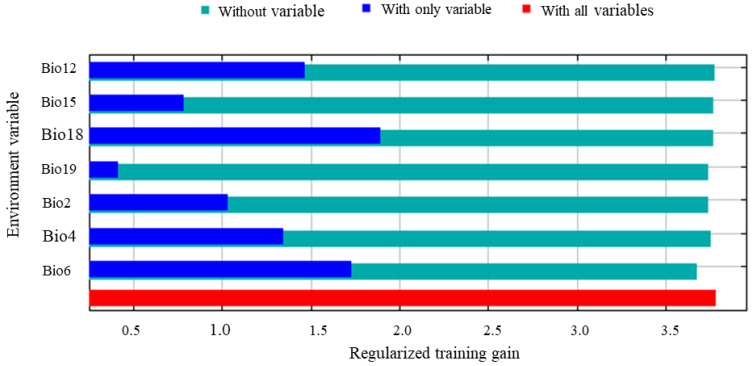
Jackknife test of the main environmental variables for *Stephanitis chinensis*.

In the normalized training gain of dominant environmental variables, a longer bar indicates that the variable, when used independently, provides more informative value than others and exerts a stronger influence on species distribution (Figure 5). Among the seven selected environmental variables, the mean precipitation of the warmest quarter (Bio18) and the minimum temperature of the coldest month (Bio6) exhibited the highest training gains. Annual precipitation (Bio12) and temperature seasonality (Bio4) also showed considerable contribution. The mean diurnal temperature range (Bio2) and precipitation seasonality (Bio15) provided moderate improvement to the model’s performance, whereas the mean precipitation of the coldest quarter (Bio19) contributed the least, suggesting that it contained limited useful information. Regarding the decrease in training gain after removing each variable, Bio6 showed the largest reduction, indicating that this variable carries unique and irreplaceable predictive information for the potential distribution of *S. chinensis*. In terms of variable contribution and permutation importance, Bio18 and Bio4 contributed most substantially, with 48.9% and 24.7%, respectively, while Bio6 contributed 14.2% and exhibited the highest permutation importance (87.7%) (Table 1). Collectively, these results demonstrate that Bio18, Bio6, Bio12, and Bio4 are the dominant environmental variables shaping the distribution pattern of *S. chinensis*.

Bio6 has the highest permutation importance and Bio18 has the highest contribution rate, which may be because *S. chinensis* has a single host and is highly dependent on tea plants. Both precipitation and low temperature significantly affect the survival and growth of tea plants, which in turn influence the feeding and reproduction rates of *S. chinensis*. In addition, *S. chinensis* is a temperate and subtropical insect with weak cold resistance; the minimum temperature of the coldest month directly determines its overwintering survival rate, and low temperatures can lead to widespread death of its eggs and nymphs. A suitable range of 454–672 mm is consistent with the characteristics of mean precipitation in the warmest quarter in the subtropical and temperate humid regions, where *S. chinensis* naturally distributes in China. The humidity conditions within this range can meet its growth and development needs, while excessively high or low precipitation will inhibit its population size.

### 3.3. Response Curves of Key Environmental Variables

**Figure 6:**
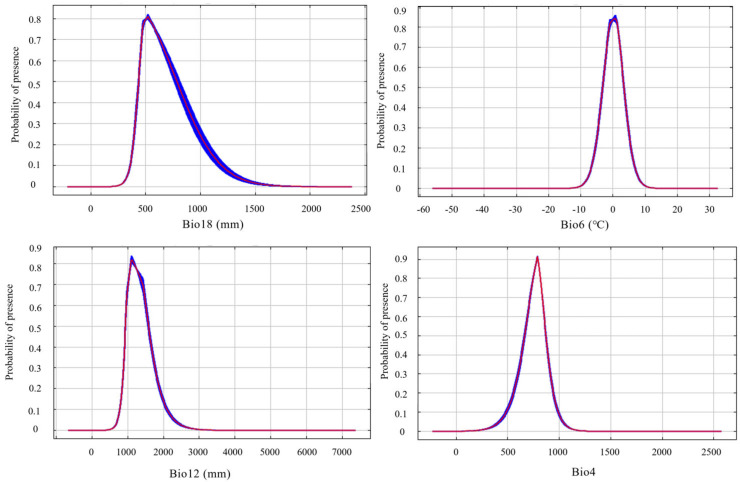
The response curves of the main environmental variables for *Stephanitis chinensis*.

According to the response curves of the dominant environmental variables generated by the MaxEnt model, a probability of occurrence greater than 0.6 was considered indicative of the suitable range for each variable. The probability of *S. chinensis* occurrence increased with rising mean precipitation of the warmest quarter, reaching a peak at approximately 521 mm and remaining suitable within the range of 454–672 mm; beyond this range, suitability declined as precipitation continued to increase. Similar unimodal trends were observed for the minimum temperature of the coldest month, annual precipitation, and temperature seasonality. Their respective suitable ranges were −2.6–2.8 °C, 970–1500 mm, and 690–850, with peak probabilities occurring at 0.6 °C, 1115 mm, and 788 (Figure 6).

### 3.4. Potential Suitable Distribution of S. chinensis in China and Worldwide Under Current Climate Scenario

**Figure 7:**
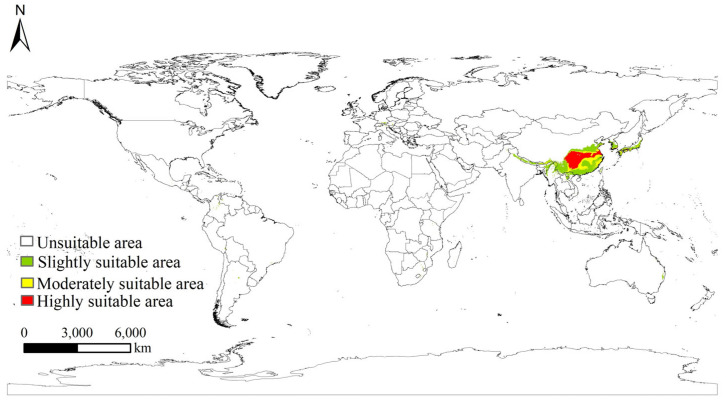
Global potential suitable habitat distribution of *Stephanitis chinensis* under current climatic scenarios.

Under current climate conditions, the potential suitable distribution of *S. chinensis* covers 2.08% of the global land area, excluding Antarctica (Figure 7). The species is primarily concentrated in eastern and southern Asia, while only scattered and fragmented suitable patches occur along the coastal regions of southern Europe, southeastern North America, and southeastern South America, without forming continuous large-scale suitable zones. Globally, highly suitable, moderately suitable, and slightly suitable habitats account for 1.18%, 0.41%, and 0.49% of land area, respectively. Highly suitable habitats are overwhelmingly concentrated in China, with only small isolated highly suitable patches found in southern Japan and southern Korea, mostly confined to warm and humid localities and lacking extensive continuous distribution. China thus represents the core distribution region of *S. chinensis*, with a substantially larger suitable area, population density, and invasion risk than other parts of the world. Consequently, analyses of future climate scenarios in this study primarily focus on projected changes in the potential distribution of *S. chinensis* within China.

**Figure 8:**
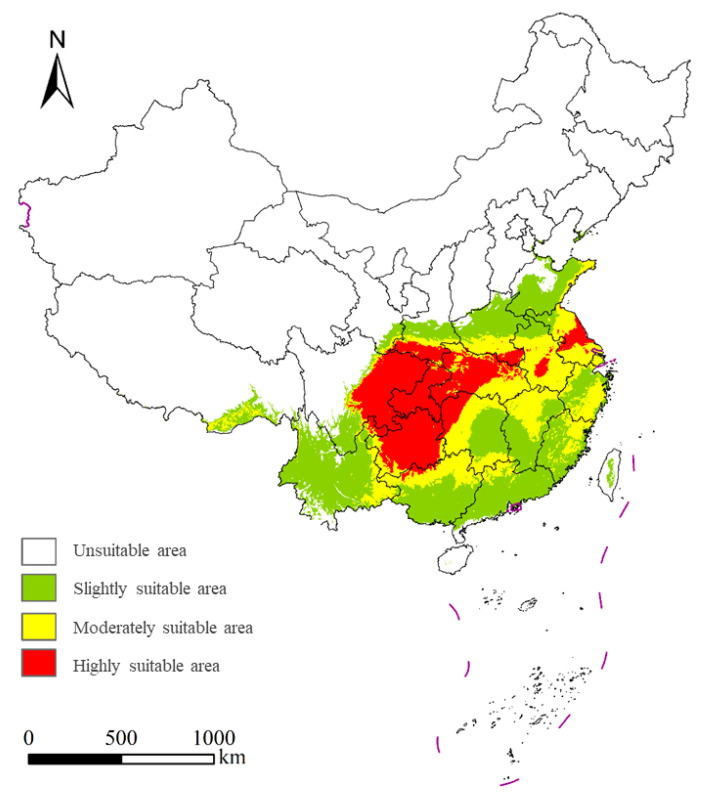
Potential distribution regions of *Stephanitis chinensis* under the current climate scenarios in China.

The potential suitable habitat of *S. chinensis* encompasses 28.58% of the total land area of China (Figure 8). Highly suitable habitats (6.48% of China) are mainly concentrated in Guizhou, Chongqing, eastern Sichuan, Hubei, northwestern Hunan, southern Shaanxi, and central Jiangsu, with small additional patches in northern Yunnan, central Anhui, southern Henan, and northern Guangxi. Moderately suitable habitats (8.14%) are primarily distributed across Anhui, Jiangsu, Zhejiang, Hunan, Hubei, northern Jiangxi, southern Henan, southern Shaanxi, northeastern Fujian, northern Guangdong, northern Guangxi, and eastern Yunnan. Additional fragmented patches occur along the Shandong coast, southeastern Tibet, Sichuan, northeastern Chongqing, Guizhou, and southern Gansu. Slightly suitable habitats (13.96%) are mainly found in Yunnan, Guangdong, Guangxi, Shandong, Zhejiang, Fujian, central Hunan, central-to-southern Jiangxi, northern Henan, central Shaanxi, northern Anhui, and southern Sichuan. Small patches also occur in southeastern Tibet, western Guizhou, southeastern Gansu, Taiwan, and northwestern Jiangsu, with scattered occurrences in parts of Chongqing, Hubei, Hainan, Hebei, and Liaoning.

### 3.5. Potential Distribution of S. chinensis in China Under Future Climate Scenarios

**Figure 9:**
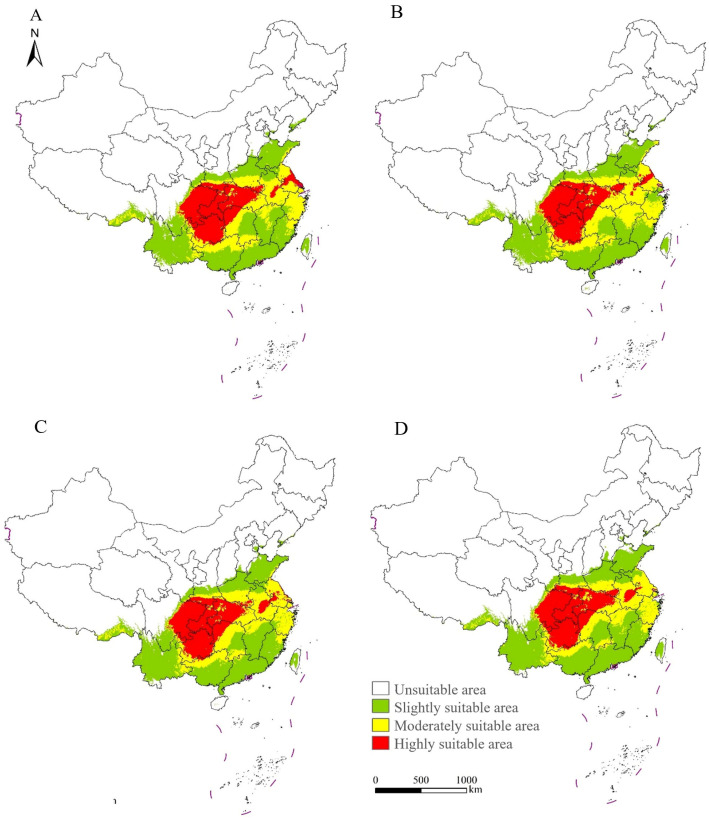
Projected suitable regions of *Stephanitis chinensis* under future climate scenarios in China. (**A**) SSP126 in the year 2050. (**B**) SSP126 in the year 2070. (**C**) SSP585 in the year 2050. (**D**) SSP585 in the year 2070.

The potential distribution of *S. chinensis* was projected for 2050 and 2070 under two climate scenarios, SSP126 and SSP585 (Figure 9). Across all future scenarios, the current suitable habitats will largely be retained, and highly suitable habitats will remain concentrated in Guizhou, Chongqing, eastern Sichuan, Hubei, northwestern Hunan, and southern Shaanxi, forming a continuous distribution belt. Under the SSP126 scenario, highly suitable habitats will expand mainly into Hubei and the coastal areas of Jiangsu by 2050; moderately suitable and slightly suitable habitats will show only minor changes. Some slightly suitable zones in Zhejiang will gradually shift to become moderately suitable. By 2070, highly suitable areas will extend further into Hubei and southern Henan, and additional slightly suitable regions in Zhejiang and Jiangxi will transition into moderately suitable regions. Under the SSP585 scenario, highly suitable habitats will expand primarily into central Anhui and Hubei by 2050. By 2070, further expansion will occur in Hubei, central Anhui, and southern Henan. In contrast, highly suitable habitats in Jiangsu will show a noticeable contraction, with the reduction in 2070 being greater than that in 2050. Meanwhile, slightly suitable habitats in Zhejiang will progressively shift to become moderately suitable, whereas some moderately suitable regions in Guangdong, Guangxi, Fujian, and Jiangxi will become slightly suitable. Across both climate scenarios, areas currently classified as unsuitable are projected to transition into slightly suitable habitats, mainly in Shandong, Shanxi, Hebei, Tianjin, and Taiwan.

### 3.6. Changes in Suitable Habitat Area and Centroid Shifts in S. chinensis Under Climate Change Scenarios

**Figure 10:**
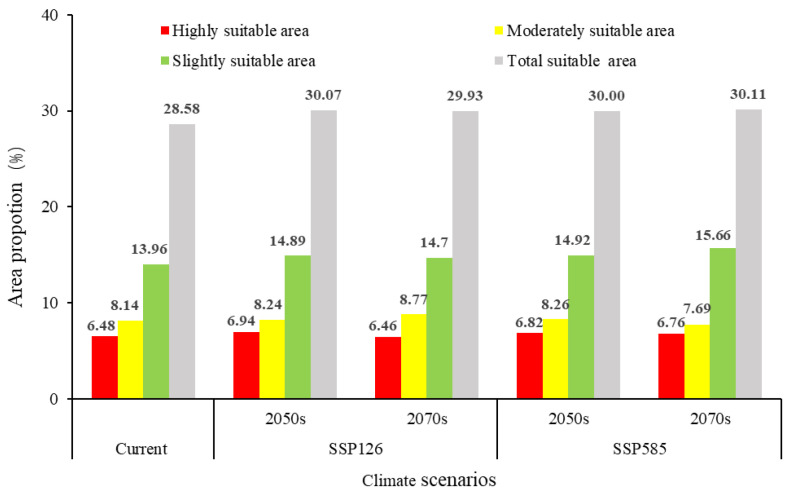
Potentially suitable distribution regions of *Stephanitis chinensis* under different climate scenarios in China.

**Figure 11:**
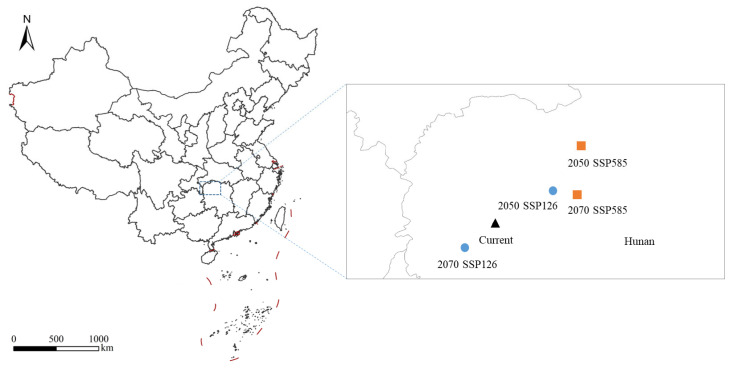
The centroid migration of *Stephanitis chinensis* in China under different climate scenarios.

Under the SSP126 and SSP585 climate scenarios, the total potential suitable area for *S. chinensis* in China is projected to increase to varying degrees (Figure 10). Under SSP126, the total suitable area will expand by 1.49% by 2050, with simultaneous increases across highly, moderately, and slightly suitable zones. By 2070, the overall suitable area will increase by 1.35%; although the extent of highly suitable habitats is projected to show a slight decline, the contraction is minimal, and both moderately and slightly suitable zones will continue to expand. Under SSP585, the total suitable area will increase by 1.42% by 2050, accompanied by increases across all suitability levels. By 2070, the suitable area will increase by 1.53%, representing the largest projected gain among all scenarios. During this period, highly and slightly suitable habitats will continue to expand, whereas the moderately suitable zone will exhibit a contraction. Notably, in both climate scenarios, the area classified as highly suitable in 2070 is projected to be smaller than in 2050, suggesting that emerging environmental conditions may reduce the survival probability of *S. chinensis* within these zones over time. Spatially, the migration distances of the habitat centroids under the 2050 SSP126, 2070 SSP126, 2050 SSP585, and 2070 SSP585 scenarios are 53.31, 32.27, 96.13, and 67.11 km, respectively. All centroids fall within Hunan Province. With the exception of a southwestward shift under 2070 SSP126, centroid movement in all other scenarios is directed toward the northeast (Figure 11).

## 4. Discussion

This study represents the first application of the MaxEnt model to predict the potential suitable distribution of *S. chinensis* in China. Across both current and future climate scenarios, the model yielded AUC values exceeding 0.99, indicating “excellent” predictive performance. Previous studies have shown that when sample sizes exceed 90, MaxEnt models achieve high accuracy and stability, with the standard deviation of AUC values remaining below 0.05, thereby ensuring robust and reliable predictions [17]. In the present study, 98 occurrence records of *S. chinensis* were employed, satisfying the sample size requirement for accurate modeling. MaxEnt has become one of the most widely used species distribution models in recent years, frequently producing more precise predictions compared with alternative approaches [18,19,20].

An evaluation of environmental variables revealed that the mean precipitation of the warmest quarter (Bio18), minimum temperature of the coldest month (Bio6), annual mean precipitation (Bio12), and temperature annual range (Bio4) are the dominant factors shaping *S. chinensis* distribution. Each factor exerts a specific regulatory effect on *S. chinensis* survival and reproduction through different ecological pathways. Within defined ranges, both the mean precipitation of the warmest quarter and annual mean precipitation exhibit a unimodal effect on the species’ presence probability. Specifically, precipitation values of 454–672 mm during the warmest quarter and 970–1500 mm annually provide favorable conditions for the survival of *S. chinensis*, closely corresponding to the optimal precipitation range for tea plant (*Camellia sinensis*) growth. Excessive or insufficient precipitation will inhibit its feeding and oviposition; meanwhile, this precipitation range is highly consistent with the optimal water requirement for tea tree growth, indirectly providing a stable food source and habitat for *S*. *chinensis*, thus forming a “climate–host” coordinated adaptation mechanism. Temperature also plays a crucial role in shaping the geographic distribution and overwintering survival of *S. chinensis*. Mean temperatures exceeding 28 °C during decadal periods adversely affect development, while the minimum temperature of the coldest month directly determines overwintering success. This study found that *S. chinensis* thrives when the minimum temperature of the coldest month is between −2.6 and 2.8 °C and the temperature annual range is 690–850. Regions in central and western China satisfy these conditions, featuring complex topography and a combination of subtropical monsoon and mid-subtropical climate characteristics. The warm and humid environment of these regions closely matches the species’ ecological requirements. Furthermore, these areas coincide with ecologically suitable zones for tea cultivation, providing a high degree of climate–host plant matching that facilitates *S. chinensis* survival and reproduction.

In terms of species interaction and habitat adaptation within China, the potential suitable areas of *S. chinensis* largely overlap with those of *E*. *onukii* [14,15], *Euproctis pseudoconspersa* [16], and *Ectropis grisescens* [21], covering most tea cultivation regions. However, the spatial patterns of suitability differ substantially among these species, likely due to differences in genetic characteristics, ecological niche differentiation, thermal tolerance, and environmental adaptation strategies [22,23,24,25,26,27]. The spatial overlap of *S. chinensis* with other major tea pests may induce synergistic damage effects, thereby increasing potential ecological threats to China’s tea industry. Under future climate scenarios, the species’ slightly suitable areas are projected to expand toward higher latitudes, while moderately suitable areas exhibit a composite pattern of southern contraction and northern advancement. This suggests that warming climates may reduce habitat suitability in southern regions due to high-temperature stress, whereas newly suitable areas in northern regions may provide ecological space for population expansion. This northward expansion is consistent with trends observed for multiple tea pests [14,15,16,21] and aligns with the general pattern of latitudinal shifts in insect distributions under global warming [22,27]. It also reflects the adaptive response mechanism of tea lace bugs to climate change. In addition, the expansion of highly suitable areas in Hubei, Anhui, Henan, and other regions is closely related to local climate change and the distribution of tea planting, and the two have synergistically promoted the population expansion of this species. Field survey data in 2024 (severe outbreaks occurred in the Wuhan and Shiyan regions of Hubei Province, and this insect was monitored in the Xinyang region of Henan Province) further verified this mechanism. In primary tea plantations, natural enemies such as *Stethoconus japonicas* Schumacher can regulate population growth, whereas newly invaded plantations, lacking natural enemies, are prone to outbreaks [28]. Consequently, in highly suitable areas where *S. chinensis* has already been established, the conservation and utilization of natural enemies should be prioritized, while unsurveyed tea-growing regions require strengthened monitoring and pre-emptive pest management strategies.

In this study, seven environmental variables were selected to predict the potential distribution of *S. chinensis* under current and future climate conditions. However, species distribution is influenced not only by abiotic climatic factors but also by biotic interactions, including mutualism, competition, and predation, as well as by species dispersal capacity and geographic characteristics. The relative importance of these factors varies across spatial scales [10,29,30,31]. The host plant of *S. chinensis* is relatively limited, being primarily restricted to tea. Studies at the agricultural landscape scale have demonstrated that enhancing the ratio of predatory natural enemies to pests can effectively stabilize pest populations [32,33]. Currently, areas in China that are highly or moderately suitable for tea planting account for 86.84% of the existing tea-planting area [34]. Therefore, future research should integrate the spatial distributions of host plants and natural enemies and comprehensively account for multiple environmental factors to improve the accuracy of species distribution models. The integration of pest population genetics and ecological niche modeling has established a “gene–environment–geography” research framework, which serves as a key approach for predicting pest invasion pathways, assessing prevention and control risks, and developing precise management strategies [35,36,37].

## 5. Conclusions

Based on 98 occurrence records of *S. chinensis* and seven key environmental factors, the global and Chinese occurrence patterns of this species have been summarized. Climatic factors such as the mean precipitation of the warmest quarter predominately influence its distribution, and its suitable hydrothermal conditions match those of tea plant growth, forming a climate–host coordinated adaptation mechanism, with core suitable areas in central and western China. Its suitable areas overlap with those of other major tea pests, possibly causing synergistic damage. Under future warming, low-suitability areas will expand northward, while moderately suitable areas will contract southward and advance northward. The lack of natural enemies triggers outbreaks in newly invaded tea plantations; thus, natural enemy conservation should be prioritized in highly suitable areas and monitoring should be enhanced in uninvestigated tea regions. Future studies should integrate multiple factors and combine population genetics with ecological niche modeling to improve prediction accuracy and support the precise management of *S. chinensis*.

## Figures and Tables

**Table 1:** Percentage contribution and permutation importance of environmental variables.

Variable	Significance of Variables	Percent Contribution/%	Permutation Importance/%
Bio18	Whether hydrothermal conditions are synchronized	48.9	1.1
Bio4	Average temperature and its variation range	24.7	0.8
Bio6	Impact of extreme temperatures	14.2	87.7
Bio15	Precipitation amount and its seasonal distribution	6.8	0.3
Bio2	Characteristics of temperature difference	3	3.3
Bio19	Whether hydrothermal conditions are synchronized	1.9	5.8
Bio12	Variation in annual precipitation	0.6	1

## Data Availability

The original contributions presented in this study are included in the article/Supplementary Material. Further inquiries can be directed to the corresponding author.

## Supplementary Materials

The following supporting information can be downloaded at: https://www.mdpi.com/article/10.3390/insects17030279/s1, Table S1: Locations of *Stephanitis chinensis*.
